# Rare TACI Mutation in a 3-Year-Old Boy With CVID Phenotype

**DOI:** 10.3389/fped.2019.00418

**Published:** 2019-10-15

**Authors:** Lucia Leonardi, Giulia Lorenzetti, Rita Carsetti, Simona Ferrari, Alessia Di Felice, Bianca Cinicola, Marzia Duse

**Affiliations:** ^1^Division of Pediatric Immunology and Rheumatology, Department of Pediatrics, Sapienza University of Rome, Rome, Italy; ^2^B Cell Physiopathology Unit, Immunology Research Area, Bambino Gesù Children Hospital, Rome, Italy; ^3^Department of Medical Genetics, Policlinico S. Orsola-Malpighi, Medical University of Bologna, Bologna, Italy

**Keywords:** CVID phenotype, TNFRSF13B, C193X, RAG1, LIG1

## Abstract

Common variable immunodeficiency (CVID) is the most common and clinically relevant primary immunodeficiency (PID). Genetic basis of CVID remains largely unknown. However, in a minority of CVID patients, a number of distinct genetic defects affecting the normal processes of B cell maturation and differentiation into memory B cells have now been identified, resulting in markedly reduced serum levels of immunoglobulin G (IgG) and low immunoglobulin A (IgA) or immunoglobulin M (IgM), with impaired antibody responses, despite the presence of normal levels of B cells. Patients with CVID develop recurrent and chronic infections of respiratory and gastrointestinal tracts, autoimmune diseases, lymphoproliferative complications, malignancies, and granulomatous disease. We report the case of a boy admitted to our unit for the first time at the age of three for reduced gamma globulin levels and a clinical history positive for two episodes of pneumonia. Our patient incompletely met ESID diagnostic criteria for CVID, but molecular genetic analysis, a NGS panel including 47 PID-associated genes was performed in the proband and in his parents, revealing the presence of a heterozygous nucleotide substitution in exon 4 (c.579C>A) of *TNFRSF13B* encoding TACI. This mutation has been described only in two CVID adult patients and in a child with selective IgA deficiency (sIgAD). We highlighted the same mutation in the asymptomatic mother and detected two extra heterozygous mutations of *RIG1* and *LIG1*. We promptly started intravenous immunoglobulin (IVIG) therapy with good tolerance. Despite the diagnosis of CVID remains clinical, in this case report we underline the importance of considering and planning genetic workup in all subjects with unclear diagnosis and of reporting new molecular diagnosis especially in case of rare mutations.

## Background

Hypogammaglobulinemia in children is sustained by a large spectrum of clinical conditions. Transient hypogammaglobulinemia of infancy (THI) should be considered during the first years of life, due to age dependent maturation of adaptive immunity. THI is characterized by immunoglobulin G (IgG) below age-related normal value in absence of defined causes of hypogammaglobulinemia, detected in the first 3 years of life (measured at least twice), with spontaneous resolution approximately after the 4th birthday. In children in the first 3 years of life with low IgG levels the definition of unclassified antibody deficiency (UNPAD) should be considered, reserving THI for those children who recover by the age of four. To rule out THI, a definite diagnosis of common variable immunodeficiency (CVID) should not be given before the age of four (1). However, alternative diagnosis for unclassified antibody deficiency includes early-onset CVID.

CVID is the most common primary immunodeficiency (PID) affecting about 20% of all PIDs patients in Europe and 25% of CVID patients are diagnosed in childhood (2, 3). Despite the majority of CVID cases seems to occur sporadically, about 5 to 25% of patients have a positive family history and a monogenic defect has been identified in 2–10% of patients with CVID phenotype (2). However, the current opinion is that, apart from these rare monogenic forms, CVID is more likely a complex rather than a Mendelian disease and modifier genes may play a crucial role in the development of disease (1). The spectrum of TACI mutations is becoming broader as more patients are screened worldwide. *TNFRSF13B*/TACI defects represent, indeed, the most common DNA sequence variations in individuals affected by CVID, being found in about 10–20% of subjects (4) and associated with CVID specific phenotypes, including the presence of benign lymphoproliferation and autoimmunity. After the initial description of an association with both CVID and selective immunoglobulin A deficiency (sIgAD) (5, 6), further analyses have shown the existence of some *TNFRSF13B* variants also in healthy individuals (7) and unaffected family members of patients with CVID (8–11). In addition, the functional effects of *TNFRSF13B* mutations in relation to the development of the disease have not been completely established yet and seem to include both causal and modifier mutations.

## Case Report

We report the case of a boy admitted to our unit for the first time at the age of three for reduced gamma globulin levels and a clinical history positive for two episodes of pneumonia.

**Figure 1:**
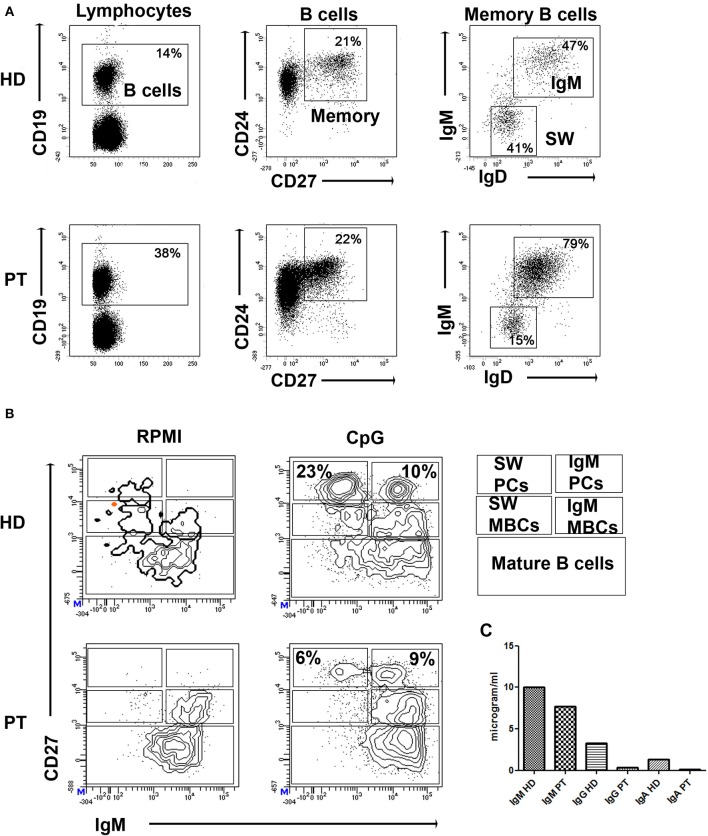
**(A)** Dot plots show the gates used to identify B cells (CD19^pos^), CD24^pos^CD27^pos^ memory B cells (MBCs), and inside MBCs the IgM^pos^ and IgM^neg^ (SW). **(B)** PBMCs were kept in culture (RPMI) or stimulated *in vitro* with CpG for 7 days (CpG). Staining with CD27 and IgM identifies IgM MBCs (IgM^pos^CD27^pos^, indicated as IgM MBCs) and switched MBCs (IgM^neg^CD27^pos^, indicated as SW MBCs). Plasma cells have higher levels of CD27 and express either IgM (IgM PCs) or switched isotypes (SW PCs). CD27^neg^IgM^pos^ cells are mature B cells. **(C)** Levels of secretory immunoglobulin in cell medium of HD and patient (PT) after stimulation with CpG.

The patient was born by cesarean section after a full-term uncomplicated gestation. He was admitted at kindergarten at 1 year of age and completed a regular immunization schedule. His parents were non-consanguineous and family history was negative for PIDs, but positive for coeliac disease (sister), allergic rhinitis (paternal grandmother), atopic asthma (mother/father). The child had a clinical history of bronchitis with wheezing since the age of 4 months. He had no known allergies. At the age of 29 months he was diagnosed with bronchopneumonia involving the right lower lobe. Seven months later he was admitted to our pediatric unit for persistent fever unresponsive to antibiotic therapy and he was diagnosed with bronchopneumonia involving the right middle lobe. Routine blood tests showed low serum immunoglobulin for his age: IgG 330 mg/dl (n.v. > 462); IgA 20 mg/dl (n.v. >27) and IgM 69 mg/dl (n.v >62). Cell blood count and lymphocyte subsets assay as well as IgG subclass were in the normal range, while anti-tetanus IgG (0.1 UI/ml) and anti-pneumococcal IgG (0.9 mg/l) were not protective despite previous vaccination in the first year of life. Booster doses of tetanus and pneumococcal vaccines were administered and, after 21 days, anti-tetanus toxoid IgG and anti-pneumococcal capsular polysaccharide IgG achieved a borderline protective level (anti-tetanus IgG 0.44 UI/ml, anti-Pneumococcal IgG 36 mg/l) suggesting a potential THI. Screening for autoimmunity, including coeliac disease, was negative. In addition, it was found a positivity of EBV VCA IgM and IgG with negative EBV DNA (RT-PCR) in absence of specific clinical manifestation. An accurate follow-up was planned, including periodical evaluation of clinical conditions and laboratory findings. Three months later, clinical conditions remained good (the patient did not present severe infections or autoimmune manifestations), but immunoglobulin levels were persistently lower than the normal range and low switched memory B cells for age were detected (Figure 1) as well as persistent positivity of EBV VCA IgM and IgG with negative RT-PCR. We therefore performed an NGS PID panel covering 47 classical genes associated with humoral and combined immunodeficiencies and immune dysregulation diseases (*ADA, BTK, CD19, CD79A, IGLL1, CD79B, PIK3R1, TCF3, DCLRE1C, TNFRSF13B, CD81, MS4A1, CR2, NFKB1, NFKB2, PIK3CD, CTLA4, LRBA, CASP10, IL2RA, PTEN, RAG1, RAG2, CECR1, AICDA, CD40LG, CD40, ATM, UNG, TTC7A, IKZF1, LIG1, OAS1, DKC1, STAT1, STAT3, XIAP, SH2D1A, FAS, FASLG, CASP8, NFKBIA, PLCG2, PRKCD, PRF1, NRAS, KRAS*). The genes on the panel have been carefully selected based on scientific literature, mutations databases, and our experience.

Molecular analysis revealed a heterozygous nucleotide substitution in the exon 4 (c.579C>A) of *TNFRSF13B* (ClinVar: NA; ExAC: 0.00005), being inherited from the asymptomatic mother. This determines the substitution of a cysteine (C) with a stop codon (X) causing premature termination of the protein translation (p.C193X). Moreover, two extra heterozygous mutation were detected: *LIG1* p.Val753Met (ClinVar:NA;ExAC:0.007) inherited from the mother, and *RAG1* p.Pro1028Leu (ClinVar:NA; ExAC: 0.000008) inherited from the father who was asymptomatic as well.

Following molecular results and in consideration of the progressive decreasing IgG levels to 290 mg/dl, the proband started intravenous immunoglobulin therapy (IVIG) once a month, without any adverse effects. Nowadays the patient is in good general condition, no further major infectious episode nor autoimmunity manifestations and lymphoproliferation occurred.

Written informed consent was obtained from the patient's parents prior to the publication of this case report.

## Discussion

Our case was firstly diagnosed as unclassified antibody deficiency as alternative diagnosis of potential THI because of age of onset, low serum IgG, slightly low serum IgA, and borderline protective antibody levels after a booster injection of tetanus and pneumococcal vaccines. At the age of 39 months clinical conditions and serological values were unchanged. Our extended NGS analysis (47 PID genes) revealed the presence of a heterozygous nucleotide substitution in the exon 4 (c.579C>A) of *TNFRSF13B*. This determines the substitution of a cysteine (C) with a stop codon (X) causing the premature termination of protein translation (p.C193X). C193X variant is described in the ExAc population with a frequency of 0.00005. The analysis showed that our patient inherited this mutation from his mother, who was completely asymptomatic.

Moreover, two extra heterozygous mutation were detected: *LIG1* p.Val753Met (ClinVar:NA; ExAC:0.007) inherited from the mother, and *RAG1* p.Pro1028Leu (ClinVar:NA; ExAC:0.000008) inherited from the father who was asymptomatic as well. Both parents showed normal serum immunoglobulin, lymphocyte subsets and B lymphocytes phenotype.

Literature search for *TNFRSF13B* mutation (C193X) identified two adult patients diagnosed with CVID and a child with sIgAD. Salzer et al. (11) evaluated the genetic, immunologic, and clinical condition of 50 individuals with *TNFRSF13B* alterations: the same mutation of our patient was described in heterozygosis in a 50-year-old man with CVID and autoimmune haemolytic anemia, coeliac disease and splenomegaly. This mutation was also described by Pulvirenti et al. (12) in homozygosis in a 49-year-old CVID patient with severe B-cell lymphopenia (<1%), autoimmune haemolytic anemia, lymph nodes enlargement and granulomas. In the same article, the same mutation is described in heterozygosis in a 6-year-old child diagnosed with sIgAD (with no clinical manifestations of autoimmunity). In the Italian cohort of 189 CVID patients described by Pulvirenti et al. the C193X allelic frequency is in accordance with the Hardy-Weinberg equilibrium (12). However, the frequency of whole *TNFRSF13B* monoallelic mutations is significantly higher in CVID patients compared to healthy controls (*p* = 0.0034, as calculated using two-tailed Fisher's exact test). ClinVar did not report on the pathogenicity of this variant. Nevertheless, there are evidence in literature that TACI haploinsufficiency is involved in B cell dysfunction (13, 14). On the other hand, the existence of healthy controls with heterozygous mutations in this gene and the lack of a clear Mendelian inheritance in families have led to consider some of *TNFRSF13B* mutations as risk factors (9, 11), which could be determinant to cause disease only in homozygous individuals (6). Thus, *TNFRSF13B* could be considered a modifier gene rather than a causal gene in monoallelic cases (2).

The monoallelic *LIG1* and *RAG1* mutations detected have not been associated with CVID phenotype while *TNFRSF13B* variants are described in heterozygosis in CVID patients. Since our patient mutation is inherited from the asymptomatic mother, we speculate that this TACI mutation could be a modifier in a *RAG1-LIG1* digenic disease. However, it remains necessary to understand the functional role of these genes' mutations.

Molecular findings were crucial for the diagnosis of CVID in our patient, as ESID diagnostic criteria for CVID were not completely met (15). More specifically, our patient presented marked decrease of IgG, slightly decreased IgA with normal IgM, low switched memory B cells for his age (Figure 1), and borderline protective antibody response to vaccines after the second booster. He did not present autoimmune manifestations, granulomatous disease, unexplained polyclonal lymphoproliferation, affected family members with antibody deficiency.

A strict clinical follow up has been planned in order to promptly detect any possible evolution of the clinical picture toward autoimmune, lymphoproliferative and granulomatous disease, while IVIG was started. Clinical follow up will clarify if early introduction of IVIG therapy can slow down the evolution of the immunodeficiency. It would also be interesting to know more about the clinical history evolution of the described pediatric case carrying the same mutation and diagnosed with sIgAD.

In conclusion, current opinion is that in case of hypogammaglobulinemia in children younger than 4 years molecular investigation for CVID is not required (1). However, last version of ESID criteria may be unable to identify all CVID cases, so that many patients under 4 years of age remain undiagnosed. Therefore, considering that the spectrum of mutations causing CVID is becoming broader as more patients are screened worldwide, an extended NGS panel is useful in all suspected cases to obtain information leading to a diagnosis that anticipate complete clinical presentation and allow a prompt therapeutic intervention. In addition, we highlight the importance of reporting new molecular diagnosis in order to better understand the pathophysiology of CVID, clarifying the clinical significance of rare mutations, differentiating disease-causing alterations and disease-modifying ones.

## Data Availability Statement

All datasets generated for this study are included in the manuscript/supplementary files.

## Author Contributions

LL, GL, AD, BC, and MD wrote the first draft of the manuscript. SF performed the molecular analysis. RC performed the immunological functional study. LL and GL wrote the final version of the manuscript. All authors contributed to manuscript revision, read, and approved the submitted version.

### Conflict of Interest

The authors declare that the research was conducted in the absence of any commercial or financial relationships that could be construed as a potential conflict of interest.
